# Patient safety in dentistry – a decade in the making

**DOI:** 10.1038/s41415-025-8384-1

**Published:** 2025-05-23

**Authors:** Edmund Bailey, Mohammed Dungarwalla

**Affiliations:** 121113547048040582250 Reader/Honorary Consultant in Oral Surgery, Institute of Dentistry, Queen Mary University of London, United Kingdom; 936796042643357238997 Clinical Lecturer/Specialist in Oral Surgery, Institute of Dentistry, Queen Mary University of London, UK and Royal London Hospital, Whitechapel, London, E1 1FR, United Kingdom

## Abstract

Patient safety is a fundamental aspect of any healthcare system. We explore the development of patient safety both generally and in relation to dentistry over the past ten years. Other aspects of healthcare and various concepts are explained and described, including human factors, Safety I and Safety II, patient safety culture, managing patient safety incidents and the second victim concept, perfectionism and punishment myths, and hierarchy, along with wellbeing and support for practitioners. We bring together ten years of experience in patient safety related to dentistry and discuss this in the context of wider developments in patient safety, with reference to reports and policies that have influenced this field. We also include helpful resources and suggestions to allow readers to discover more about patient safety in dentistry, and to examine the safety culture in their own organisations. We finish this paper by contemplating on what the next decade might bring.

## Key points

- Explores the various concepts and initiatives which have been developed to maintain and improve patient safety in dentistry and healthcare, including NHS England's 2024 ‘Primary care patient safety strategy', which specifically mentions primary care dentistry.
- The authors reflect on the past ten years of working in the field of patient safety in dentistry and look ahead to the next ten years, which is likely to include greater use of technology and access to summary care records for clarification of medical histories.
- Patient safety solutions and resources are provided for dental professionals to make use of in their own clinical practice.

## Background

The understanding of patient safety has evolved over the past ten years in the United Kingdom (UK) and internationally. However, there are only a small number of researchers specifically looking at this topic in relation to dentistry, and much of this work has focused on extraction of the correct tooth after wrong tooth extraction was defined as a surgical ‘never event' by the NHS (National Health Service) in England in 2015. This was subsequently removed from the never events list in 2021, justified due to the systemic barriers to prevent the removal of wrong teeth not being strong enough to prevent these incidents from occurring.^1^

Although important, and arguably the most tangible patient safety incident in dentistry, there are other aspects of patient safety which require development. The use of surgical safety checklists is a fundamental part of patient safety as it helps to overcome the risks posed by human factors and certain systems failures.

A recent scoping review found that checklists are confined to oral surgery-related procedures and are still mainly used in specialist settings and academia.^2^ Furthermore, while there is evidence to support the use of checklists to minimise patient safety incidents and patient harm, especially in relation to wrong tooth extraction,^3^ it is also suggested that checklists in isolation may be ineffective.^2^ Only in combination with an environment which encourages a positive patient safety culture and the necessary team engagement and training will the efficacy of checklists become apparent.^2^^,^^4^

It is widely acknowledged that institutions with a positive patient safety culture will provide safer care to their patients. Achieving this positive culture is both multi-factorial and challenging. This paper marks the ten-year anniversary of the lead author's first published paper in this field.^5^ We will summarise some of the developments over the past decade and update the reader on the state of patient safety in dentistry as of 2025, with a view to the future.

Over the past decades, the focus on patient safety within the health service has gathered in momentum.^6^ There are now several academic journals dedicated to patient safety and a plethora of policy documents and textbooks dedicated to this theme.^7^^,^^8^ One of the triggers for research into patient safety was a report from the United States (USA)^9^ published in 2000, which highlighted that as many as 98,000 people were dying each year because of medical errors occurring in hospitals. It is thought that if patient safety incidents were included in the league tables for causes of death, patient safety incidents, or iatrogenic harm, could be the third leading cause of death in the USA after heart disease and cancer.^10^ Up to 50% of adverse events are thought to be preventable.^11^ Furthermore, it is thought that 15% of hospital expenditure and activity in Organisation for Economic Cooperation and Development countries can be attributed to treating safety failures.^12^ Along with this fiscal cost, there are negative impacts due to patient suffering, the loss of trust in healthcare systems and the burden of this on the teams that work in healthcare.

## Defining patient safety

It is useful to revisit definitions of patient safety. This definition has evolved over the years as a result of attempts to make it encompass the complex nature of safety in healthcare. As of 2025, the most widely accepted definition is:

- ‘Patient safety is a framework of organised activities that creates cultures, processes, procedures, behaviours, technologies, and environments in healthcare that consistently and sustainably lower risks, reduce the occurrence of avoidable harm, make error less likely and reduce its impact when it does occur'.^13^

## Safety I and Safety II – moving on from the Swiss cheese?

The first author's 2014 paper focused on the Swiss cheese model and its use in explaining the factors which can lead to patient safety incidents.^5^ While this research is still valid, our understanding of this subject has developed.

**Fig. 1:**
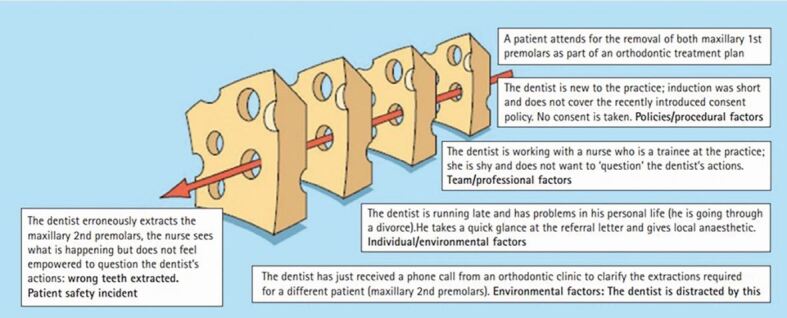
Swiss cheese model in relation to dentistry. Reproduced from Bailey *et al*., ‘Patient safety in primary care dentistry: where are we now?', *British Dental Journal*, volume 217, Springer Nature, 2014, with permission from SNCSC^5^

This much cited model was first proposed by James Reason in 1990.^14^ An example of this from a dentistry setting is shown in Figure 1.

This model is helpful in describing conceptually how adverse events or systems failures occur. In an ideal world, there would be several safeguards and layers of barriers and defences in place that work in tandem to prevent errors occurring. The reality is that these barriers are similar to slices of Swiss cheese in that they have potential holes in each barrier layer. The presence of holes in one barrier layer is unlikely to lead to an error as the other barrier layers should prevent this. When the holes in each of the barrier layers line up, they form a trajectory of accident opportunity, and it is highly likely that an error or incident will occur in these circumstances.^5^^,^^15^

This model, although extremely useful, and adaptable to demonstrate why incidents occur, may not always be appropriate for health-related incidents. The model is linear in nature and not always applicable. It assumes that adverse outcomes can be identified and fixed, usually through compliance, standardisation of process and training. There is an assumption that when errors occur, it is due to a deviation from standard, successful care. This is not always the case and there is now a body of evidence on the interactions between the parts of a system which are interdependent (clinicians, patients and equipment/environment). This approach is often termed ‘Safety II'. ‘Safety Iʹ was concerned with studying events retrospectively and trying to learn from these infrequent occurrences. Safety II aims to focus on why things go well and to examine the resilience in systems that are working effectively, acknowledging that the vast majority of outcomes are successful.^8^^,^^16^ Table 1 summarises some of the differences between Safety I and Safety II.

**Table 1:** Safety I and Safety II approaches

	**Safety I**	**Safety II**
Definition	Things only go wrong on rare occasions	Most things go to plan
Humans/workforce	Can be seen as a hazard/liability	Necessary resource
Management principle	Reactive approach. Responds to incidents	Proactive, anticipates events and considers the impact that changes will have
Purpose of investigation	Identify the causes of failings	Understand what works well in order to explain why things occasionally go wrong
Risk assessment	Identify causes from investigations/contributary factors	Understands that performance variability in complex systems is difficult to monitor/control

## Human factors

The opening line from Gorovitz and MacIntyre's 1976 piece, ‘Toward a theory of medical fallibility' reads, ‘no species of fallibility is more important or less understood than fallibility in medical practice'.^17^

Almost five decades after this piece was written, while the narrative and acceptance of fallibility have been incorporated into legal and ethical frameworks of medical practice, the premise is strikingly similar: clinicians make mistakes, and unfortunately, this can and has resulted in harm and suffering.

It is important to briefly explore some of the natural reactions to errors in clinical practice – these are also set out in the NHS England ‘Patient safety strategy'.^18^ Firstly, challenging or attempting to rectify processes and/or clinicians responsible for the error is unlikely to result in meaningful and lasting change. Also known as the ‘bad apple fallacy', attempting to root out individuals implicated in medical error may temporarily address the incident and provide respite to those negatively affected by the outcomes; however, it is no guarantee that the error will not recur, and it certainly does not address the often complex, multifactorial nature of how the error occurred in the first place.

Secondly, is to address the ‘perfection myth'. While practising a task many times can improve quality and outcomes, persistent training in a particular task or process does not guarantee the incident will not occur again. Furthermore, following a patient safety incident or medical error, should training be targeted to specific individuals, or should training be targeted to an entire team or division? Does the training address the complexity of interactions and processes that took place at the time of the incident?

Factors such as fatigue, poor wellbeing, hubris, overconfidence and ego contribute to medical error. Box 1 shows some brief acronyms which may be used to describe the circumstances in which staff might become unsafe.

As this paper focuses on the dental team, some of these factors are expanded in the subsequent sections. This is not an exhaustive list and these factors should not be considered in isolation but as a continuum of each other.

The authors describe the type of errors related to human factors in the original piece.^5^

Box 1 Two useful acronyms used to describe the circumstances in which staff might become unsafe

- Hungry Angry Late Tired (HALT)
- Illness Medication Stress Alcohol Fatigue Emotions (IMSAFE)

## Addressing the hierarchy and authority gradients

It is not uncommon for parallels to be drawn between healthcare and the aviation industry.^19^^,^^20^^,^^21^^,^^22^

While many similarities exist, there are key differences between healthcare and aviation, primarily, the potential large-scale burden to human life in the latter and pilots often falling directly victim to the consequences of their decision-making.

Crew resource management (CRM) emerged in aviation to address the non-technical skills pilots were expected to possess.^19^ One aspect of CRM is empowering junior pilots and crew members to participate in and significantly intervene in decisions made by senior pilots/captains that were perceived to affect operations negatively.

In a hospital setting, most clinical mistakes occur in the operating theatre.^19^ The combination of multiple staff who may be unacquainted with one another, with the often stressful and time-pressured task of complex surgery, are factors leading to clinical errors in this environment. It is now well-recognised that all team members have a right to voice concerns when they believe patient safety is compromised. However, in practice, its implementation may be variable.

Korean Air Cargo 8509 was a scheduled flight that departed from London Stansted Airport in December 1999. Shortly after take-off, the captain, a very experienced former military pilot, received incorrect and misleading flight observations on his instrument panel due to faulty sensory equipment. By contrast, the much more junior first officer received the correct readings on his flight instrument display, yet failed to speak out to counteract the captain's retaliatory manoeuvres to the incorrect data he received on the instrument display. The plane struck the ground, claiming the lives of all crew on board. The investigation cited that the junior first officer may have ‘felt inhibited in bringing the situation to the attention of the commander'.^23^

This raises the question of how to address hierarchy in clinical teams. Hierarchy itself may not be problematic; it can offer patients a degree of reassurance that a senior clinician is involved in their care, and it can reassure junior clinicians that they will be supported, supervised and trained as part of their professional development.

**Fig. 2:**
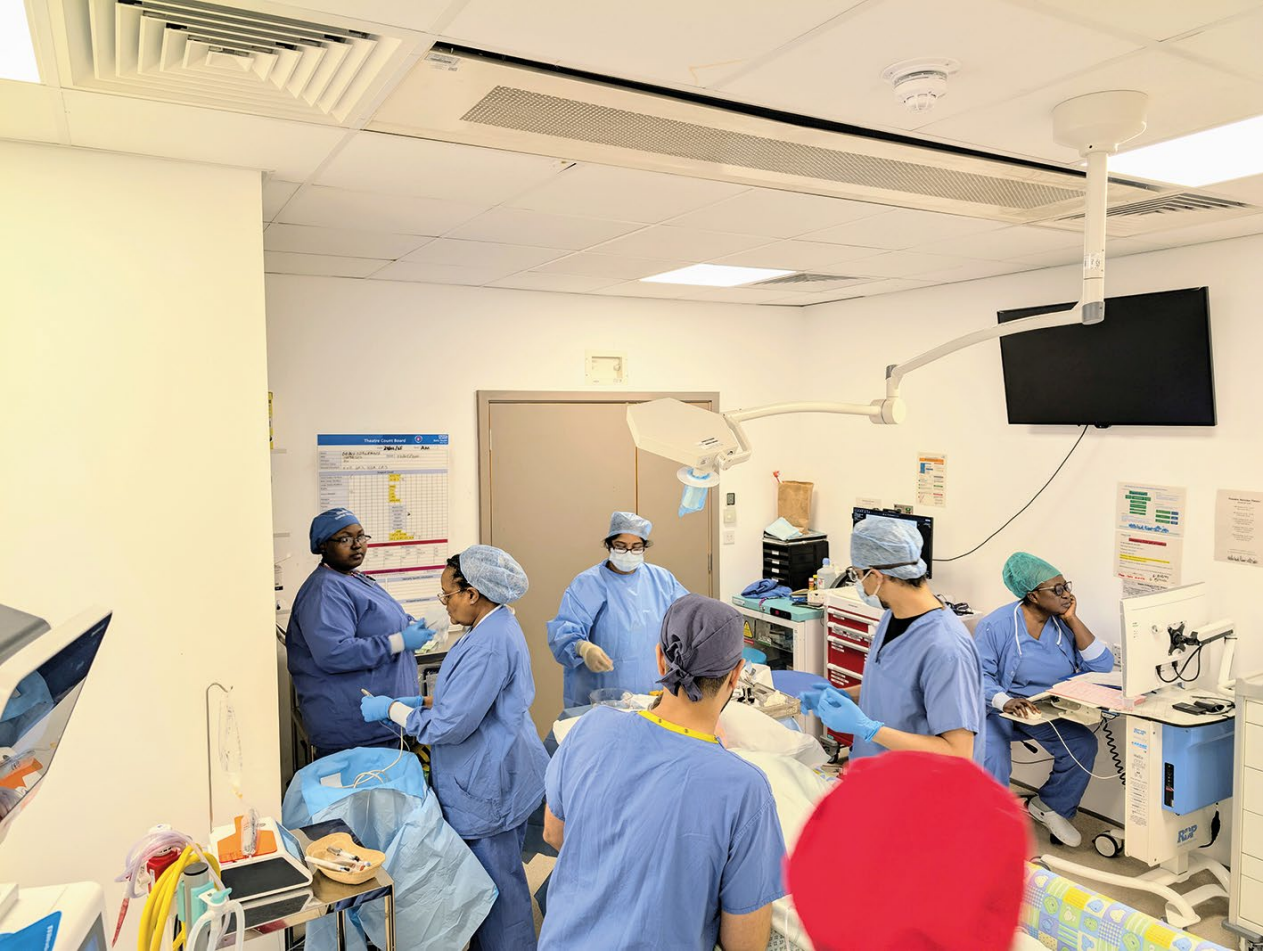
Who is who? A busy theatre team, some of whom have never worked with one another before. Similar outfits can lead to confusion and reluctance to speak up, particularly if team members are unaware of names and roles

**Fig. 3:**
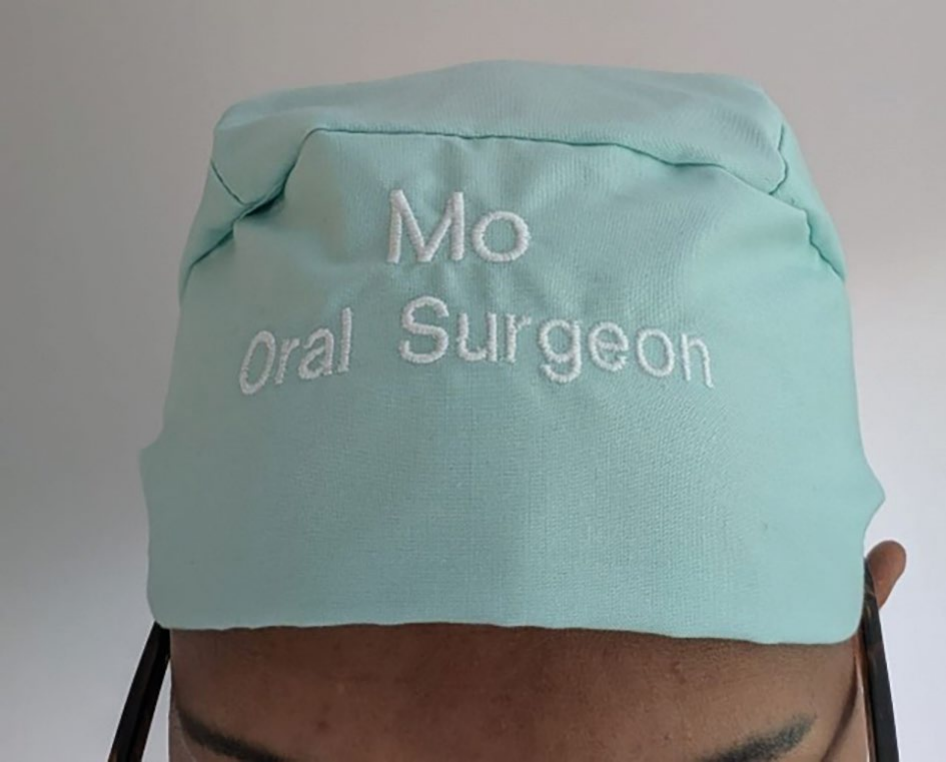
A personalised cap made available to all team members outlining the name and role. These small aides can be extremely useful when addressing team members in a busy environment with many unfamiliar faces

Hierarchy can be problematic when other team members fail to speak out when they perceive that patient safety or quality is compromised. To overcome this, the authors recommend and implement the following strategies in their practice: namely, dedicated induction talks discussing the concept of speaking up and not being afraid of reprimand; engaging in theatre briefs and morning huddles, and engaging all team members in this exercise, ensuring that names and roles of clinicians are visible throughout the day to ensure that unacquainted and unfamiliar team members can quickly address one another (Fig. 2, Fig. 3); and recommending junior clinicians plan complex cases and discuss these with more senior clinicians before surgery so that more junior team members have contributed to the planning and delivery of the case.

In a primary care setting, pre-clinic huddles can serve a similar purpose. They allow team members to discuss their roles for the session. They also provide an opportunity to discuss matters that will affect the running of the clinical session, for example, the types of cases, the materials required and availability of materials and equipment, staff absence, and travel issues that may affect patient attendance. NHS England has published ‘huddle sheets', available online, which provide a tool to discuss and reflect on matters which may affect patient safety.^24^ The tool provides examples in the following domains:

- People and teams
- Tasks
- Tools and equipment
- Working environment
- Organisation
- External factors.

## Fatigue, wellbeing and burnout

These factors have been given prominence, particularly since the COVID-19 pandemic. Data show that in 2022, NHS England experienced an absence rate of 5.6%, which has often been attributed to burnout.^25^

The same report notes that 170,000 NHS staff have left or are considering leaving the service due to workload and stress.^26^

Burnout is a term which originated in the 1970s. However, its use has become more prominent in healthcare settings, compounded by challenges precipitated by the COVID-19 pandemic. Burnout describes emotional, physical and mental exhaustion caused by excessive and prolonged stress.^27^

Importantly, clinical teams and management should be aware of how burnout can affect individuals and, as a result, clinical teams and patient outcomes.

The NHS staff wellbeing needs poster, published by NHS Employers in 2022,^28^ is adapted from Maslow's hierarchical needs, which outlines the requirements for NHS staff to feel healthy at work. This document divides needs into three groups: basic, psychological and self-fulfilment.

Basic needs indicate the essential resources for staff, including nutrition, hydration, adequate and appropriate clothing, including personal protective equipment, toilet and sanitary facilities, and provisions for breaks.

Psychological needs include the ability to speak and ensure one's voice is heard by senior leadership and management (this is discussed later), being recognised as a valued team member, being rewarded for one's work, and having a meaningful personal development plan.

Self-fulfilment needs include the ability to support and inspire others, deliver excellent patient care, and develop within one's role.

Notably, the needs described above are not exhaustive and are open to modification depending on the individual, team and environment to which they are being applied. For example, training junior dentists and dental undergraduates in the authors' clinical work setting forms a large proportion of both workload and clinical time. Ensuring the appropriate resources, time and faculty (longer appointments, dedicated chairside supervision, dedicated feedback facilities, and student welfare facilities) are available to deliver excellent-quality clinical teaching is a stipulation of the above self-fulfilment needs. These factors are not necessarily as important or relevant to settings where teaching does not feature in the working day.

These factors may also be far more difficult to apply in primary care settings, where the environment may be geared towards managing high patient turnover due to growing demand and backlogs of care.

There must be processes in place to allow clinicians who are at risk of becoming unsafe to gain support and mitigation of these feelings.

## Patient safety culture

For patient safety initiatives to be effective, it is essential that the appropriate culture is prevalent in the clinical setting. A positive or generative patient safety culture is one where safety is prioritised, resourced and practised by the team and its leaders. Table 2 summarises these qualities and behaviours in relation to a dental practice setting.

**Table 2:** Qualities and behaviours that may be considered ‘functional' or ‘dysfunctional' in a primary dental care setting. These may also be considered as ‘pathological' or ‘generative'

**Dysfunctional**	**Functional**
Inappropriate blame/isolating blame to one individual	Using a ‘systems' approach to drive improvement
‘We've always done things this way'	‘We value any suggestions which would improve quality of care'
Ignoring incidents	Learning from incidents and sharing learning via a team discussion
Concerns are ignored or brushed aside	Concerns are treated seriously and acted upon through a structured framework
Silo working/individual working	Responsibilities are shared
Poor communication/lack of communication streams	Communication between individuals and the wider team is straightforward
Individual approaches ignore stakeholders when designing a service	Participatory design
Potential risks are ignored	Systems are designed to minimise risk, and potential risks are identified, shared and managed with a team approach

The NHS England ‘Patient safety strategy' was published in 2019.^18^ The strategy encourages an engaged, visible leadership, promoting openness, just culture and continuous improvement, valuing diversity and equality. The document states that in a positive patient safety culture, safe care is delivered through:

- ‘Continuous learning and improvement of safety risks
- Supportive, psychologically safe teamwork
- Enabling and empowering speaking up by all'.^18^

The NHS England ‘Just culture guide' can be used by employers where there is suspicion that an incident occurred due to the potentially negligent actions of an individual who will then require specific support or interventions to enable them to work safely. This may include involving regulatory bodies. This will rarely be required; however, reasonable consideration of this aspect of safety needs to be part of any healthcare system with a positive safety culture.

Work on improving patient safety culture has been carried out using a Safety II approach. The team looked at organisations which were identified by the Care Quality Commission as being rated as ‘good' or ‘outstanding' for ‘safe' and then looked at what their good practices were.

Psychological safety involves:

- Civility – making personal connections with team members
- Creating a leadership promise and behaviour framework that staff can sign up to
- Appreciation of team members and granting them the permission and freedom to innovate.

Patient safety culture in dentistry appears to lag compared to medical specialties, especially in general dental practice. This may be due to several reasons:^29^

- Dentistry is lower risk than medicine/surgery
- The outpatient nature of dentistry makes following-up on complications and care-related issues challenging
- Data collection can be difficult due to variations in dental care records
- Around 90% of dental care is carried out in dental practices which are run as businesses and reporting of harms may have a commercial/financial impact.

There are very few publications on patient safety culture in dentistry, and those that are published tend to focus on secondary care dental settings.^6^^,^^30^ However, there is qualitative evidence that dental teams have high levels of knowledge and experience in maintaining patient safety.^31^^,^^32^^,^^33^

Building a patient safety culture in dentistry:

- Encourages an open culture where all members of the team feel empowered to speak up on issues
- Brings safety to the agenda during meetings
- Encourages incident reporting, even for incidents considered to be low harm or near misses
- Investigates safety incidents using a transparent process
- Learns from these investigations and adopts a ‘just culture' and supportive processes for managing those involved
- Provides training for the team on patient safety and non-technical skills for surgeons
- Moves from a reactive culture to a proactive one, where systems are designed with safety in mind, rather than waiting for incidents to occur and reacting to these.

When incidents do occur, the incident has an impact on the patient and their family primarily; these are the first victims. The clinician or clinical team involved in the incident can suffer psychological harm because of the incident and are termed second victims.^34^ The ‘second victim' concept has caused some controversy in the literature. While it is important to support healthcare workers who are involved in safety incidents, using the term ‘victim' suggests no responsibility for the incident, and no accountability in dealing with the consequences. Being a ‘victim' also suggests passiveness and lack of agency. For these reasons, some patients and their families avoid the use of the word ‘victim' in relation to their own experiences of safety incidents.^35^

**Fig. 4:**
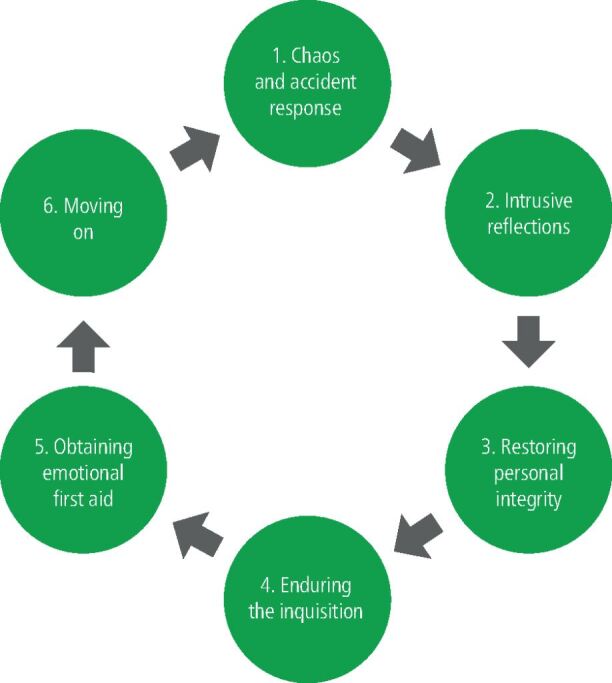
The six-stage cycle that occurs in the aftermath of an event

A six-stage cycle is described that occurs in the aftermath of an event (Fig. 4).

Emotional and psychological support can help second victims to progress through this cycle more rapidly.^36^^,^^37^ Some of the behaviours observed in those who are second victims include:^38^

- Hypervigilance Stress Anxiety Shame
- Feelings of inadequacy
- Risk avoidance
- Self-doubt about knowledge and skills
- Insomnia
- Difficulty concentrating.

Furthermore, the impact on clinicians, especially in training, can be immense. It can also increase the risk of future incidents if not properly managed. This can lead to underperformance and depression, leading to a detrimental effect on patient safety. Early intervention and support are recommended to minimise the long-term sequela.

The Patient Safety Incident Response Framework (PSIRF) was rolled out during 2023–2024 and is a contractual requirement for NHS trusts. Primary care settings may wish to adopt PSIRF; however, it is not mandatory. This new framework includes a focus on engaging with everyone affected by patient safety incidents, and a document titled ‘Engaging and involving patients, families and staff following a patient safety incident' is being developed. The process for reporting incidents is now known as ‘learn from patient safety events' (LFPSE). Datix and similar incident report systems now feed into this. These efforts are part of a culture change away from the previous ‘blame culture' into a more supportive culture which has been observed in healthcare settings.

Unfortunately, in dentistry, a culture of fear seems to prevail in terms of incident reporting and the perceived punitive consequences, which may include litigation and regulatory action associated with being involved in an incident.^6^^,^^31^ This has also been highlighted as a problem with medical doctors, who are often reluctant to report incidents.^39^ These are aspects of the ‘punishment myth' – the idea that if we punish people when they make errors, they will not make them again.

We hope that the adoption of the NHS England ‘Just culture guide' may help with managing those involved in safety incidents in a more supportive fashion while still highlighting areas of malpractice which do require intervention from an employer or regulatory body.

The 2019 NHS England ‘Patient safety strategy' has recently been supplemented by the ‘Primary care patient safety strategy', which was launched in September 2024. This strategy specifically mentions primary dental care. Among the aims of the strategy are:

- To provide access to the National Care Records Service for dentists in primary care. This provides the summary care record (current diagnoses, medications and allergies) and other important health-related information, such as child protection information, reasonable adjustments, mental health plans, transfer of care plans and end-of-life care plans
- To allow access to dental teams to the LFPSE national reporting system for incident reports
- To identify patient safety themes in dentistry and develop and test novel approaches for improvement and sharing of good practice
- Dental staff to complete local staff surveys, with action taken on the findings of these.

Allowing dentists to access the National Care Records Service will be a progressive step towards maintaining and improving patient safety and quality of care.^40^ Relying on patient-declared medical histories before treating patients is fraught with problems. Patients often present to dental practices with several comorbidities and polypharmacy, and a proportion of patients will not fully recall their medical and drug histories.^41^ This can be more of an issue with certain vulnerable groups, including those with dementia and those with English as a second language. It can also be an issue when seeing emergency patients where no prior records are available. When dentists are provided with this information from the National Care Records Service, there may be a reduction of referrals to secondary care, as the practitioner will be more confident to manage the patient when they are fully informed of their medical history.

Project Sphere is a recent initiative by NHS England and the Office of the Chief Dental Officer. This project is specifically referenced in the ‘Primary care patient safety strategy'. The project aims to develop safety initiatives in dentistry and to deal with the blame culture which is so often cited. In June 2023, the group published huddle sheets, which have already been discussed.

## Freedom to speak up

As discussed above, the freedom to speak up forms one of the psychological components of staff wellbeing needs.

In the last ten years, the UK has been rocked by high-profile health scandals, several of which have identified failures resulting from staff raising concerns to senior colleagues and being ignored. The most notable of these took place at the Countess of Chester Hospital NHS Foundation Trust, where neonatal nurse Lucy Letby was found guilty of murdering seven newborn babies.^42^ It is reported, however, that concerns about Letby were first raised in 2015 by a doctor, and no action was taken.^43^

All NHS organisations have ‘freedom-to-speak-up guardians'. These independent bodies are responsible for maintaining safety and quality of care and for providing confidential support to staff who cannot escalate concerns via conventional routes.

## The current landscape and the next ten years

At the time of writing, improving access to NHS dentistry was a significant election talking point for several political parties. There are ample stories of ‘DIY (do it yourself) dentistry', with members of the public suffering considerably from poor oral health.^44^ This lack of access to dental care poses a great risk to patient safety, especially in light of an increased incidence of oral cancer being reported.^45^

Several strategies have been proposed or initiated to increase the uptake and scope of NHS dentistry. Namely, allowing dental hygienists and therapists to prescribe and administer a broader range of medications independently,^46^ consulting on a scheme mandating newly qualified dentists to work in the NHS for a minimum period,^47^ providing provisional registration to overseas applicants while they are awaiting complete registration with the General Dental Council,^48^ and the increasing of overseas registration examination spaces by 30%.^49^

It is not uncommon for many dental practices to have ‘in-house' specialists. Patient demands for predictable and high-quality treatment, and the continued pressure of litigation, may force even very experienced general dental practitioners to refer treatment to clinicians with specialist registration.

Interestingly, with the exception of endodontics and periodontics, the number of registrants on all specialist registers fell in 2024 when compared to 2023.^50^

Simultaneously, we are now faced with the exponentially growing market of facial aesthetics offered alongside conventional dental treatments, dental tourism, and increased use of cone-beam computed tomography to plan treatments, but largely the same number of dental and maxillofacial radiologists. The implications to patient safety in these contexts may be entering a new era, demanding changes in legislation and curricula to meet the changing face of the dental industry.

Given the changing landscape of dentistry, the question of how patient safety is incorporated into undergraduate curricula and early postgraduate training remains.^30^^,^^51^ Further work is needed on what undergraduates and early career dentists perceive as patient safety and how this impacts their training as dentists.^52^ Song *et al.* have demonstrated that gamified-enhanced patient safety in dentistry teaching has improved early career dentists' knowledge of the topic.^53^

While it is apparent patient safety remains a universal responsibility, there is still evidence to suggest that patient safety in dentistry is perceived as adhering to rules made by senior faculty.^54^ Established processes, such as incident reporting, surgical safety checklists, escalating concerns to other practitioners, identifying students and trainees with specific learning needs, and access to mental health first-aiders, all contribute to safer care.

In the authors' experience, induction talks outlining exception reporting and time off in lieu for early career trainees empower trainees to not work beyond their means and discourages the ‘grin and bear' mentality of working in busy hospital posts. This may not necessarily translate to primary care settings, where practitioners may be ferociously working to meet targets and indeed will have complete autonomy of their diary, meaning they can omit or shorten break times to continue working.

The authors note that the medical and surgical fields appear to be ahead in terms of patient safety interventions and in some cases, culture. This may be due to a perception that dental treatments are less invasive and thus carry lower risks,^4^^,^^55^ an unclear algorithm of how to report incidents to formal reporting systems, particularly in primary care settings,^55^ and a reluctance to speak up due to fear of reprimand from patients and regulatory bodies. Yansane *et al*., in a cross-sectional study that used surveys on patient safety culture to compare medical specialities and dentistry, found that improvements are required in the following domains within dentistry:

- Work pressure and pace
- Leadership support for patient safety
- Discussion and openness with regards to error.^29^

## Conclusions

The understanding of patient safety in healthcare has evolved over the past decade, with greater appreciation of the complex nature of healthcare systems and processes, along with the psychological aspects of safety moving away from both the perfection and punishment myths. In hospital dental services, surgical safety checklists are now commonplace with staff and students engaged in the process. This is not the case in primary care. There is also an acknowledgement that patient safety in dentistry is about far more than the use of checklists alone. We acknowledge that poor dental access poses a threat to patient safety in dentistry, and we welcome initiatives to improve this. We hope that the coming decade will bring further improvements and more dentistry-specific innovations, making use of ever-advancing technology. We are positive in this hope, given the work of Project Sphere and the inclusion of primary dental care in the 2024 ‘Primary care patient safety strategy' from NHS England.

## Further reading and resources

- Non-Technical Skills for Surgeons (NoTSS) from The Royal College of Surgeons of Edinburgh: https://www.rcsed.ac.uk/professional-support-development-resources/learning-resources/non-technical-skills-for-surgeons-notss
- Dental Non-Technical Skills Masterclass from The Royal College of Surgeons of Edinburgh: https://www.rcsed.ac.uk/events-courses/dental-non-technical-skills-dents-masterclass-increasing-patient-safety-how-to-assess-the-non-technical-skills-of-dentists-using-dents#EdinburghRCSEd
- Practitioner Health provides support and therapy for doctors and dentists: https://www.practitionerhealth.nhs.uk/
- Project Sphere: https://www.england.nhs.uk/primary-care/dentistry/leading-the-change/patient-safety/
- NHS England's website on patient safety: https://www.england.nhs.uk/patient-safety/
- NHS England primary care patient safety strategy: https://www.england.nhs.uk/long-read/primary-care-patient-safety-strategy/
- NHS patient safety syllabus (requires an account and login via NHS email): https://www.e-lfh.org.uk/programmes/patient-safety-syllabus-training/.
